# Prophage-encoded gene *VpaChn25_0734* amplifies ecological persistence of *Vibrio parahaemolyticus* CHN25

**DOI:** 10.1007/s00294-022-01229-z

**Published:** 2022-01-22

**Authors:** Yingwei Xu, Lianzhi Yang, Yaping Wang, Zhuoying Zhu, Jizhou Yan, Si Qin, Lanming Chen

**Affiliations:** 1 Key Laboratory of Quality and Safety Risk Assessment for Aquatic Products on Storage and Preservation (Shanghai), Ministry of Agriculture and Rural Affairs of the People’s Republic of China, College of Food Science and Technology, Shanghai Ocean University, Shanghai, 201306 China; 2 Department of Internal Medicine, Virginia Commonwealth University/McGuire VA Medical Centre, Richmond, VA USA; 3 College of Fishers and Life Science, Shanghai Ocean University, Shanghai, 201306 China; 4 Key Laboratory for Food Science and Biotechnology of Hunan Province, College of Food Science and Technology, Hunan Agricultural University, Changsha, 410128 China

**Keywords:** *Vibrio parahaemolyticus*, Foodborne pathogen, Prophage, Gene deletion and reversion, Secretome, Transcriptome

## Abstract

*Vibrio parahaemolyticus* is a waterborne pathogen that can cause acute gastroenteritis, wound infection, and septicemia in humans. The molecular basis of its pathogenicity is not yet fully understood. Phages are found most abundantly in aquatic environments and play a critical role in horizontal gene transfer. Nevertheless, current literature on biological roles of prophage-encoded genes remaining in *V. parahaemolyticus* is rare. In this study, we characterized one such gene *VpaChn25_0734* (543-bp) in *V. parahaemolyticus* CHN25 genome. A deletion mutant *ΔVpaChn25_0734* (543-bp) was obtained by homologous recombination, and a revertant *ΔVpaChn25_0734*-com (543-bp) was also constructed. The *ΔVpaChn25_0734* (543-bp) mutant was defective in growth and swimming mobility particularly at lower temperatures and/or pH 7.0–8.5. Cell surface hydrophobicity and biofilm formation were significantly decreased in the *ΔVpaChn25_0734* (543-bp) mutant (*p* < 0.05). Based on the in vitro Caco-2 cell model, the deletion of *VpaChn25_0734* (543-bp) gene significantly reduced the cytotoxicity of *V. parahaemolyticus* CHN25 to human intestinal epithelial cells (*p* < 0.05). Comparative secretomic and transcriptomic analyses revealed a slightly increased extracellular proteins, and thirteen significantly changed metabolic pathways in the *ΔVpaChn25_0734* (543-bp) mutant, showing down-regulated carbon source transport and utilization, biofilm formation, and type II secretion system (*p* < 0.05), consistent with the observed defective phenotypes. Taken, the prophage-encoded gene *VpaChn25_0734* (543-bp) enhanced *V. parahaemolyticus* CHN25 fitness for survival in the environment and the host. The results in this study facilitate better understanding of pathogenesis and genome evolution of *V. parahaemolyticus*, the leading sea foodborne pathogen worldwide.

**Supplementary Information:**

The online version contains supplementary material available at 10.1007/s00294-022-01229-z.

## Introduction

*Vibrio parahaemolyticus* is a Gram-negative bacterium that resides in aquatic environments worldwide (Letchumanan et al. 2014; Su and Chen 2020). Ingestion of raw, undercooked, or mishandled seafood contaminated by pathogenic *V. parahaemolyticus* can cause acute gastroenteritis in humans and even death (Ghenem et al. 2017; Baker-Austin et al. 2018). The bacterium was first identified from semidried juvenile sardines in 1950 in Japan, which caused 272 cases of acute diarrhea and 20 death (Fujino et al. 1953). Afterward, outbreaks of human gastroenteritis caused by *V. parahaemolyticus* occurred in many Asian nations and consequently around the world (Elmahdi et al. 2016; Meparambu Prabhakaran et al. 2020). It was estimated that more than 50% of foodborne gastrointestinal-Vibriosis cases were caused by *V. parahaemolyticus* in the United States (Karan et al. 2021), which led to 45,000 illnesses each year (https://www.cdc.gov/Vibrio/, accessed on 26 April 2021). Sporadic outbreaks have also been reported in coastal European nations (Meparambu Prabhakaran et al. 2020). China is one of the 15 countries with high incidence of diarrhea disease (Walker et al. 2013). Recently, a national surveillance of 152,792 patients of all ages with acute diarrhea was administered in 217 hospitals and 93 reference laboratories in 31 provinces of China by the Chinese Center for Diseases Control and Prevention in 2009‒2018 (Wang et al. 2021a). Total 13 common bacterial pathogens were tested, among which *V. parahaemolyticus* ranked third and contributed to 10.83% of all positive detection. Pathogenic *V. parahaemolyticus* strains produce thermostable direct hemolysin (TDH) and/or the TDH-related hemolysin (TRH) (Leoni et al. 2016; Cai and Zhang 2018). However, some clinical *V. parahaemolyticus* strains do not produce the two major toxins but display virulence, indicating that putative toxic determinants exist. Their pathogenicity might be achieved with different strategies employed by different strains (Li et al. 2020). Therefore, it is imperative to identify such risk factors in *V. parahaemolyticus* to assure food safety and human health.

Phages are found most abundantly in aquatic environments worldwide (Tan et al. 2021). Temperate phages can integrate into bacterial chromosomes with carried genes and drive bacterial genome evolution (Garin-Fernandez and Wichels 2020; Nuidate et al. 2021; Wendling et al. 2021). *Vibrio* spp. virulence-associated genes can be combined at high frequencies by horizontal gene transfer (HGT), leading to the emergence of pandemic or pathogenic clones (Castillo et al. 2018). For example, in early 1996, an atypical outbreak of *V. parahaemolyticus* infection in India was associated with a new serotype O3:K6 that carried genetic markers *tdh*, *toxRS*_new_, and *orf8*. Dispersal of this new pathogenic clone has been reported in many countries around five continents, suggesting that *V. parahaemolyticus* from the serotype O3:K6 was pandemic (Santos et al. 2021). The *orf8* gene was possibly acquired from a filamentous phage f237, which endorsed the bacterium more adhesive to host intestinal cells (Santos et al. 2021). Recently, Grain-Fernandez et al. reported a filamentous phage vB_VpaI_VP-3218 in *V. parahaemolyticus* VN-3218. It integrates into the host genome with potential zonula occludens toxin genes (Garin-Fernandez et al. 2020). Another newly isolated phage vB_VpaP_MGD2 from clam *Meretrix meretrix* was reported to be a candidate biocontrol agent against early mortality syndrome/acute hepatopancreatic necrosis disease caused by multidrug-resistant *V. parahaemolyticus* in shrimp (Cao et al. 2021).

In our previous research, *V. parahaemolyticus* CHN25 (serotype: O5: KUT) of aquatic animal origin was identified and characterized (Song et al. 2013; Sun et al. 2014; He et al. 2015; Zhu et al. 2017, 2020; Yang et al. 2020). The bacterial genome (5,443,401 bp, 45.2% G+C) contains five prophage gene clusters (6.5–36.6 kb) (Zhu et al. 2017), the largest of which showed sequence similarity to a *Vibrio* phage Martha 12B12 (Zhu et al. 2017). Within the cluster, a gene *VpaChn25_0724* (294-bp) encoding a hypothetical protein has been identified recently (Yang et al. 2020). In the present study, biological function of another prophage-encoded gene *VpaChn25_0734* (543-bp) within the same gene cluster was characterized. It showed sequence identity with components of a Phage_tail_S superfamily (Lu et al. 2020). The objectives of this study were (1) to knock out the *VpaChn25_0734* (543-bp) gene from *V. parahaemolyticus* CHN25 genome by homologous recombination to contract a deletion mutant *ΔVpaChn25_0734* (543-bp)*.* Meanwhile, a revertant *ΔVpaChn25_0734*-com (543-bp) was also constructed; (2) to examine growth, mobility, biofilm formation, and cell toxicity of the *ΔVpaChn25_0734* (543-bp) mutant compared with *V. parahaemolyticus* CHN25 wild type (WT) and *ΔVpaChn25_0734*-com (543-bp) strains; (3) to decipher the possible molecular mechanism underlying altered phenotypes of the *ΔVpaChn25_0734* (543-bp) mutant by comparative secretomic and transcriptomic analysis. The results in this study facilitate better understanding of biological function of prophage-encoded genes in *V. parahaemolyticus* genomes.

## Materials and methods

### Bacterial strains, plasmids, and culture conditions

*Vibrio parahaemolyticus* CHN25 (Song et al. 2013; Sun et al. 2014; He et al. 2015; Zhu et al. 2017, 2020; Yang et al. 2020) was stored at − 80 °C freezer in our research group. *Escherichia coli* DH5α λpir (BEINUO Biotech (Shanghai) Co. Ltd., China), *E. coli* β2155 λpir (kindly provided by Professor Weicheng Bei) and plasmid pDS132 (kindly provided by Professor Dominique Schneider) were used as a host strain for gene cloning, and a donor strain and a suicide vector for conjugation and gene knockout experiments, respectively (Zhu et al. 2017; Yang et al. 2020). Plasmid pMMB207 (Biovector Science Lab, Inc., China) was used as a gene expression vector (Zhu et al. 2017; Yang et al. 2020). *V. parahaemolyticus* and *E. coli* strains were incubated in the same media and conditions as described previously (Zhu et al. 2017; Yang et al. 2020).

### Construction of the gene deletion mutant and revertant

Gene deletion mutant and revertant of *V. parahaemolyticus* CHN25 were constructed according to the methods described previously (Zhu et al. 2017; Yang et al. 2020). Briefly, two primer pairs *VpaChn25_0734* (543-bp)-up-F/R and *VpaChn25_0734* (543-bp)-down-F/R (Table 1) were designed to target upstream (486 bp) and downstream (494 bp) sequences of the *VpaChn25_0734* (543-bp) gene in *V. parahaemolyticus* CHN25 genome, respectively. Restriction endonucleases XbaI and SacI (TaKaRa, Japan) were introduced into polymerase chain reaction (PCR) products, which were inserted into corresponding cloning sites of the pDS132. The obtained recombinant plasmid pDS132 + *VpaChn25_0734* (543-bp) was then transformed into diaminopimelic acid (DAP) auxotroph *E. coli* β2155 incubated in LB medium supplemented with 0.3 mM DAP (Sigma–Aldrich, USA) (Zhu et al. 2017; Yang et al. 2020). Double-crossover deletions of the *VpaChn25_0734* (543-bp) gene were screened using the colony PCR assay (Zhu et al. 2017; Yang et al. 2020) with the primer pair *VpaChn25_0734* (543-bp)-up-ex-F/R (Table 1). The obtained *ΔVpaChn25_0734* (543-bp) mutant was confirmed as described previously (Zhu et al. 2017; Yang et al. 2020).

**Table 1:** Oligonucleotide primers used in this study

Primer	Sequence (5′–> 3′)	Product size (bp)	References
*VpaChn25_0734* (543-bp)-up-F	GCTCTAGATGTGATCGACATCGAACAAC	486	This study
*VpaChn25_0734* (543-bp)-up-R	CTCATGGCAGTTGAGCCATCAAAGCACTCC		
*VpaChn25_0734* (543-bp)-down-F	GATGGCTCAACTGCCATGAGCCGCCCCGAT	494	This study
*VpaChn25_0734* (543-bp)-down-R	CGAGCTC CTCTTCATCGAGATACCATTG		
*VpaChn25_0734* (543-bp)-up-ex-F	CGGCGCTCTTAAAGTTCGCTC	525	This study
*VpaChn25_0734* (543-bp)-down-ex-R	GCGCGACTTCTTCTCCGTC		
*VpaChn25_0734-*com (543-bp)-F	CGAGCTCATGGCTCAAGC GGTTCGC	543	This study
*VpaChn25_0734*-com (543-bp)-R	GCTCTAGATCATGGCAGA AGCTCCTT		
*tlh-F*	AAAGCGGATTATGCAGAAGCACTG	596	Yang et al. (2020)
*tlh-R*	ACTTTCTAGCATTTTCTCTGC		
*16s RNA-F*	GACACGGTCCAGACTCCTAC	179	Yang et al. (2020)
*16s RNA-R*	GGTGCTTCTTCTGTCGCTAAC		
*VpaChn25_0734* (543-bp)*-F*	CCGGAATTC ATGGCTCAAGCGGTTCGC	543	This study
*VpaChn25_0734* (543-bp)*-R*	CCGCTCGAGTCATGGCAGAAGCTCCTT		
*VpaChn25_RS02080-F*	TGCTTGGGATAAAGACGA	1365	This study
*VpaChn25_RS02080-R*	GTAAACGGCGTGACTTCT		
*VpaChn25_RS03685-F*	TTGTAGCAGCCATGAAAG	873	This study
*VpaChn25_RS03684-R*	TATTGCGACAGCGGATAT		
*VpaChn25_RS10950-F*	ATGAAGACGGATTGGATT	1512	This study
*VpaChn25_RS10950-R*	GTGAAAGAGGAGTTGGGT		
*VpaChn25_RS11345-F*	GGTGTTGCTTGCCGTTAG	756	This study
*VpaChn25_RS11345-R*	CCGGAGGAAGAAGTTGGT		
*VpaChn25_RS13840-F*	TGATGAGACGCACAAATA	873	This study
*VpaChn25_RS13840-R*	GTAAGGATGTAACGACCAA		
*VpaChn25_RS14605-F*	TTTCACGCACTTCCACTT	963	This study
*VpaChn25_RS14605-R*	TACAGCACTATCCGAGGG		

Additionally, the *VpaChn25_0734* (543-bp) gene was obtained by PCR using the primer pair *VpaChn25_0734*-com (543-bp)-F/-R (Table 1). The PCR product was inserted into the pMMB207, and then transformed into *E. coli* DH5α. The obtained recombinant plasmid pMMB207 + *VpaChn25_0734* (543-bp) was electro-transformed into the *ΔVpaChn25_0734* (543-bp) mutant (Zhu et al. 2017; Yang et al. 2020). Positive electro-transformants *ΔVpaChn25_0734*-com (543-bp) were screened, and confirmed (Zhu et al. 2017; Yang et al. 2020). DNA sequencing was conducted by Shanghai Sangon Biological Engineering Technology and Services Co., Ltd., Shanghai, China.

### Growth curve assay

Growth of *V. parahaemolyticus* strains in the LB or TSB media at different temperatures (37 °C, 25 °C, and 15 °C) for different time (24–60 h) was individually measured using Bioscreen Automated Growth Curve Analyzer (BioTek, USA). Growth curves of *V. parahaemolyticus* strains in the TSB medium in different pH conditions (pH 5.5–8.5) were also examined, respectively (Sun et al. 2014; Zhu et al. 2017; Yang et al. 2020).

### Swimming motility and biofilm formation assays

Swimming motility of *V. parahaemolyticus* strains on semi-solid TSB (pH 8.5, 3% NaCl) agar plates (0.25% agar) was individually measured as described previously (Yang et al. 2020). The TSB agar plates were incubated at 37 °C, 25 °C, and 15 °C for 12 h, 24 h, and 72 h, respectively. Diameters at bacterial colonies were measured and recorded. Biofilm formation of *V. parahaemolyticus* strains was individually examined using the crystalline violet staining method (Yang et al. 2020). The phosphate-buffered saline (PBS, pH 7.2–7.4), and crystal violet were purchased from Sangon (China).

### Bacterial cell membrane permeability and fluidity, and surface hydrophobicity assays

Outer membrane permeability was determined according to the method by Wang et al. (Wang et al. 2021c). Briefly, a 200 μL/well of bacterial suspension in TSB medium (pH 8.5, 3% NaCl) at mid-logarithmic growth phase (mid-LGP) at 37 °C was mixed with a 2 μL/well of 10 mm *N*-phenyl-1-naphthylamine (NPN) (Sangon, China). The change of fluorescence intensity per well was measured using BioTek Synergy 2 (BioTek, USA). The excitation and emission wavelengths were 350 nm and 420 nm, respectively (Wang et al. 2021c). Inner membrane permeability was also determined (Huang et al. 2021). Briefly, a 200 μL/well of bacterial suspension was mixed with a 2.5 μL/well of 10 mM *O*-nitrophenyl-β-d-galactopyranoside (*O*-nitrophenyl)-β-d-galactopyranoside (ONPG) (Sangon, China). The mixture was incubated at 37 °C, and absorbance at OD_415 nm_ of each well was measured using BioTek synergy 2 (BioTek, USA) every 30 min (Huang et al. 2021). Cell surface hydrophobicity and membrane fluidity assays were performed as described previously (Yang et al. 2020).

### Secretome analysis

Extraction of extracellular proteins of *V. parahaemolyticus* strains grown in TSB medium (pH 8.5, 3% NaCl) at 37 °C in static culture condition, and two-dimensional gel electrophoresis (2D-GE) were performed according to the methods described previously (Zhu et al. 2020). Briefly, a 20 mg of each sample was applied to pH gradient gel strips (pH 4–7, 7 cm, Bio-Rad, Inc., USA) at 17 °C for passive rehydration for 16 h. Isoelectric focusing (IEF) for the first dimensional separation was performed using a six-step procedure, while sodium dodecyl sulfate–polyacrylamide gel electrophoresis (SDS–PAGE, 12.5% separation gels) was performed for the second dimensional separation (Zhu et al. 2020). Amino acid sequences of protein spots were determined using liquid chromatography-tandem mass spectrometry (LC–MS/MS) by Shanghai Houji Biological Co., Shanghai, China (Zhu et al. 2020).

### Human intestinal epithelial cell viability and apoptosis assay

The in vitro cell modal assay was performed according to the method described previously [26], using human rectal cancer epithelial cell line Caco-2 (ATCC number: HTB-37™, Stem Cell Bank of Chinese Academy of Sciences, Shanghai, China). Briefly, Caco-2 cells (5 × 10^4^ cells/mL per well) were cultured in Dulbecco’s modified eagle medium (DMEM, Gibco, USA) at 37 °C with 5% CO_2_ for 24 h, and then washed three times with 0.1 M PBS (pH 7.2–7.4, Sangon, China). Each of *V. parahaemolyticus* strains was grown in TSB medium (pH 8.5, 3% NaCl) at 37 °C to mid-LGP, and then harvested, washed, and resuspended in DMEM medium without phenol red. A 100 μL/well of bacterial suspension (OD_490 nm_ of about 0.2 ± 0.02), and 10 μL/well of 2-(2-methoxy-4-nitrophenyl)-3-(4-nitrophenyl)-5-(2,4-disulfonate)-2h-tetrazolium monosodium salt (CCK-8, Sigma–Aldrich, USA) were added into Caco-2 cells per well, and then incubated at 37 °C for 4 h. Caco-2 cell viability and apoptosis were examined using Annexin-V-FITC/PI Apoptosis Assay Kit (Solarbio, China) and BD FACSVerse™ flow cytometer (Becton, Dickinson and Company, USA) according to the instructions of the manufacturers (Yang et al. 2020).

### Transcriptome and data analysis

Total RNA of each of *V. parahaemolyticus* strains grown in TSB medium (pH 8.5, 3% NaCl) to mid-LGP at 37 °C were individually extracted, and purified as described previously (Yang et al. 2020). Three independently prepared RNA samples were subjected for Illumina RNA-sequencing, which was carried out by Shanghai Majorbio Bio-pharm Technology Co. Ltd., China using Illumina HiSeq 2500 platform (Yang et al. 2020). Quality filtration of raw RNA-seq data, clean read aligning, and defining of differentially expressed genes (DEGs) were conducted as described previously (Yang et al. 2020). Gene set enrichment analysis (GSEA) of DEGs was also conducted (Yang et al. 2020). Representative DEGs were examined using quantitative reverse transcription-PCR (RT-PCR) assay (Yang et al. 2020) with the primers listed in Table 1.

The data were analyzed using SPSS version 17.0 software (SPSS Inc., USA). All tests in this study were performed in at least triplicate. Phylogenetic tree was constructed using MEGA 5.0 software (Kumar et al. 2018).

### Scanning electron microscopy (SEM) analysis

Cell structure of *V. parahaemolyticus* strains grown in TSB medium (pH 8.5, 3% NaCl) to mid-LGP at 37 °C were observed and recorded using Schottky Field Emission Scanning Electron Microscope (SU5000, Japan, 5.0 kV × 35,000) at College of Food Science and Technology, Shanghai Ocean University (Shanghai, China).

## Results

### Prophage-encoded gene *VpaChn25_0734* (543-bp) in *V. parahaemolyticus* CHN25

The largest prophage gene cluster contains 24 predicted open read frames (ORFs) in *V. parahaemolyticus* CHN25 chromosome 1 (3,416,467 bp, from 816,554 to 846,961 bp) (Zhu et al. 2017). It has sequence similarity to the *Vibrio* phage Martha 12B12 (30,408 bp, GenBank accession no. NC_021070) (Yang et al. 2020). The *VpaChn25_0734* (543-bp) gene is the sixteenth ORF encodes a predicted phage virion morphogenetic protein with conserved structural domains belonging to the Phage_tail_S superfamily. In the cellular component catalogue, it was involved in constituting the intracellular membrane-bounded organelle (GO: 0043231) with a predicted GO-Score of 0.42.

### Deletion and reverse complementation of the *VpaChn25_0734* (543-bp) gene

To characterize biological roles of the prophage-encoded gene *VpaChn25_0734* (543-bp) remaining in *V. parahaemolyticus* CHN25 genome, an untagged in-frame gene deletion mutant *ΔVpaChn25_0734* (543-bp) was obtained by homologous recombination (see the Materials and Methods). The *VpaChn25_0734* (543-bp) gene was knocked out from the *V. parahaemolyticus* CHN25 genome, which was verified by PCR and DNA sequencing (data not shown), RT-qPCR, as well as transcriptome analysis (see below).

Additionally, a reverse mutant *ΔVpaChn25_0734*-com (543-bp) was also successfully obtained. The *VpaChn25_0734* (543-bp) gene was amplified, and cloned into the expression vector pMMB207, resulting in the recombinant vector pMMB207 + *VpaChn25_0734* (543-bp). This recombinant vector was electro-transformed into the *ΔVpaChn25_0734* (543-bp) mutant to obtain the revertant *ΔVpaChn25_0734*-com (543-bp), which was confirmed by the methods described above.

### Survival of *ΔVpaChn25_0734* (543-bp) at different temperatures and pH conditions

**Fig. 1:**
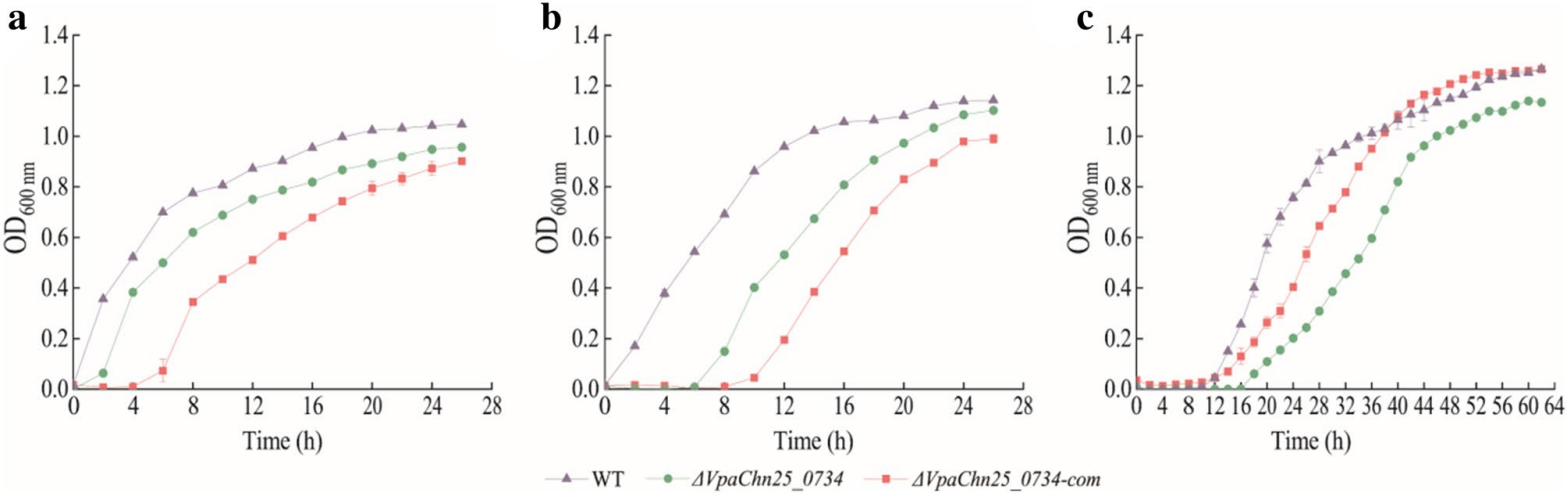
Survival of *V. parahaemolyticus* CHN25 (WT), *ΔVpaChn25_0734* (543-bp), and *ΔVpaChn25_0734*-com (543-bp) strains at different temperatures. **a**–**c** 37 °C (**a**), 25 °C (**b**), and 15 °C (**c**), respectively

To investigate the influence of *VpaChn25_0734* (543-bp) gene deletion on *V. parahaemolyticus* CHN25 survival, we determined growth curves of the WT, *ΔVpaChn25_0734* (543-bp), and *ΔVpaChn25_0734-*com (543-bp) strains at 37 °C, 25 °C, and 15 °C, which *V. parahaemolyticus* experiences during life cycle (Maje et al. 2020; Yang et al. 2020; Ali et al. 2021). At the optional growth temperature of 37 °C, the *ΔVpaChn25_0734* (543-bp) mutant grew in TSB medium (pH 8.5, 3% NaCl) with a retardation phase (RP) of 2 h, when compared with the WT strain (Fig. 1a). Similarly, at 25 °C and 15 °C, the RPs of *ΔVpaChn25_0734* (543-bp) were sixfold and 2.67-fold longer than those of WT, respectively (Fig. 1b, c). Additionally, the revertant *ΔVpaChn25_0734-*com (543-bp) appeared partially complement the defective phenotype of the *ΔVpaChn25_0734* (543-bp) mutant. These results indicated that the *VpaChn25_0734* (543-bp) gene enhanced *V. parahaemolyticus* CHN25 fitness for thriving particularly at lower temperatures.

**Fig. 2:**
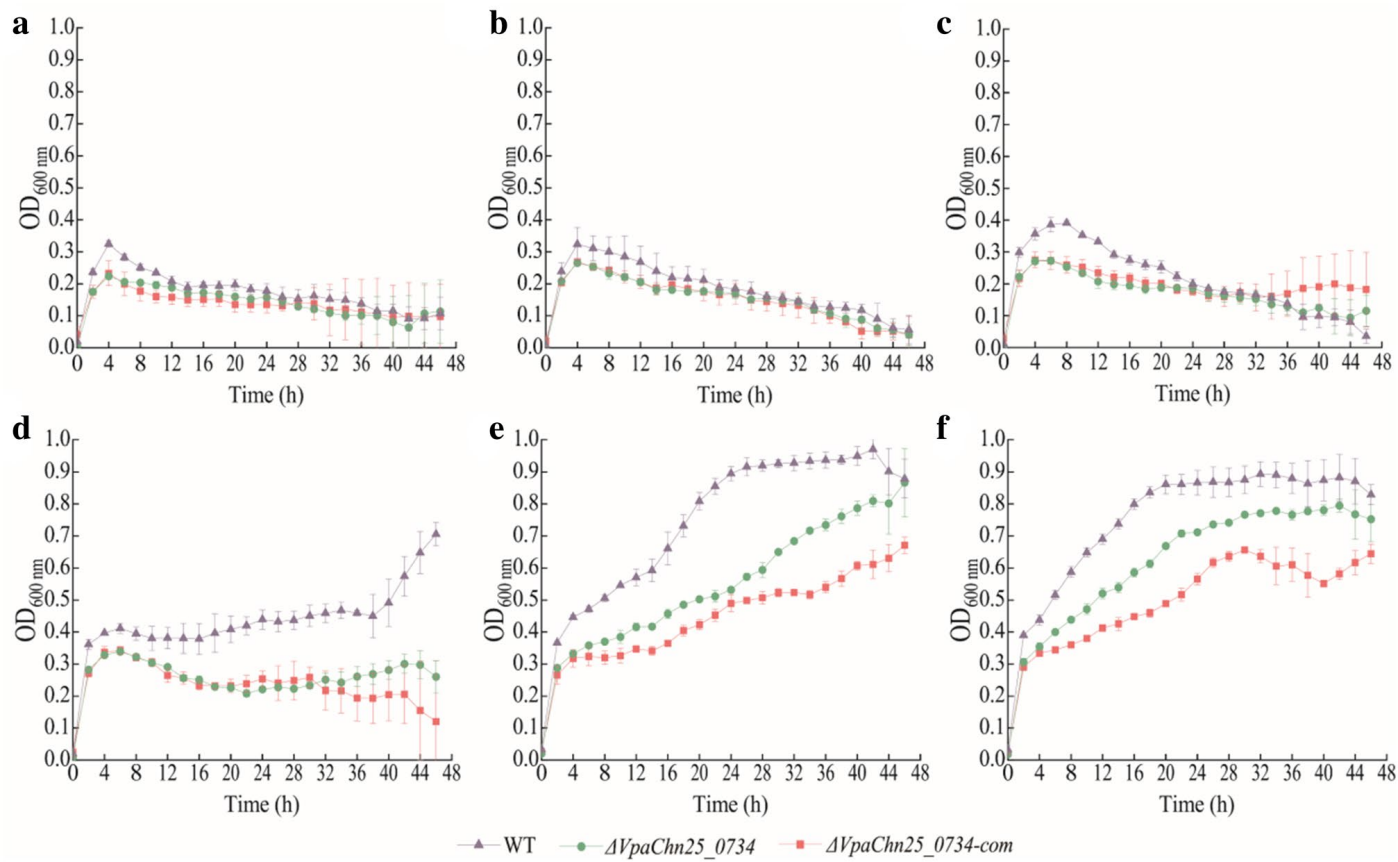
Survival of the WT, *ΔVpaChn25_0734* (543-bp), and *ΔVpaChn25_0734*-com (543-bp) strains in different pH conditions. **a**–**f** pH 5.5 (**a**), pH 6.0 (**b**), pH 6.5 (**c**), pH 7.0 (**d**), pH 7.5 (**e**), and pH 8.0 (**f**), respectively

Acid tolerance is critical for foodborne pathogens to survive against host acid gastric fluid (pH 1–3, but above 6.0 after food consumption) (Sun et al. 2014). Therefore, we examined the growth of the three strains in TSB medium (3% NaCl) with pH values in the range from 5.5 to 8.0, and the results were illustrated in Fig. 2a–f. In acidic conditions (pH 5.5–6.5), the growth of WT, *ΔVpaChn25_0734* (543-bp), and *ΔVpaChn25_0734*-com (543-bp) strains were greatly inhibited, and the maximum OD_600 nm_ values at stationary grow phage (SGP) were below 0.5 (Fig. 2a–c); in the neutral condition (pH 7.0), the growth of *ΔVpaChn25_0734* (543-bp) and *ΔVpaChn25_0734*-com (543-bp) strains were also obviously inhibited compared to the WT strain (Fig. 2d); in alkaline conditions (pH 7.5–8.0), *ΔVpaChn25_0734* (543-bp) still grew poorly with a longer RP and lower biomass than WT (Fig. 2e, f). Additionally, the plasmid-born *ΔVpaChn25_0734*-com (543-bp) did not fully complement the defective phenotype of the *ΔVpaChn25_0734* (543-bp) mutant. The results indicated that the *VpaChn25_0734* (543-bp) gene amplified *V. parahaemolyticus* CHN25 environmental persistance at pH 7.0–8.0.

### Swimming mobility of the *ΔVpaChn25_0734* (543-bp) mutant

**Fig. 3:**
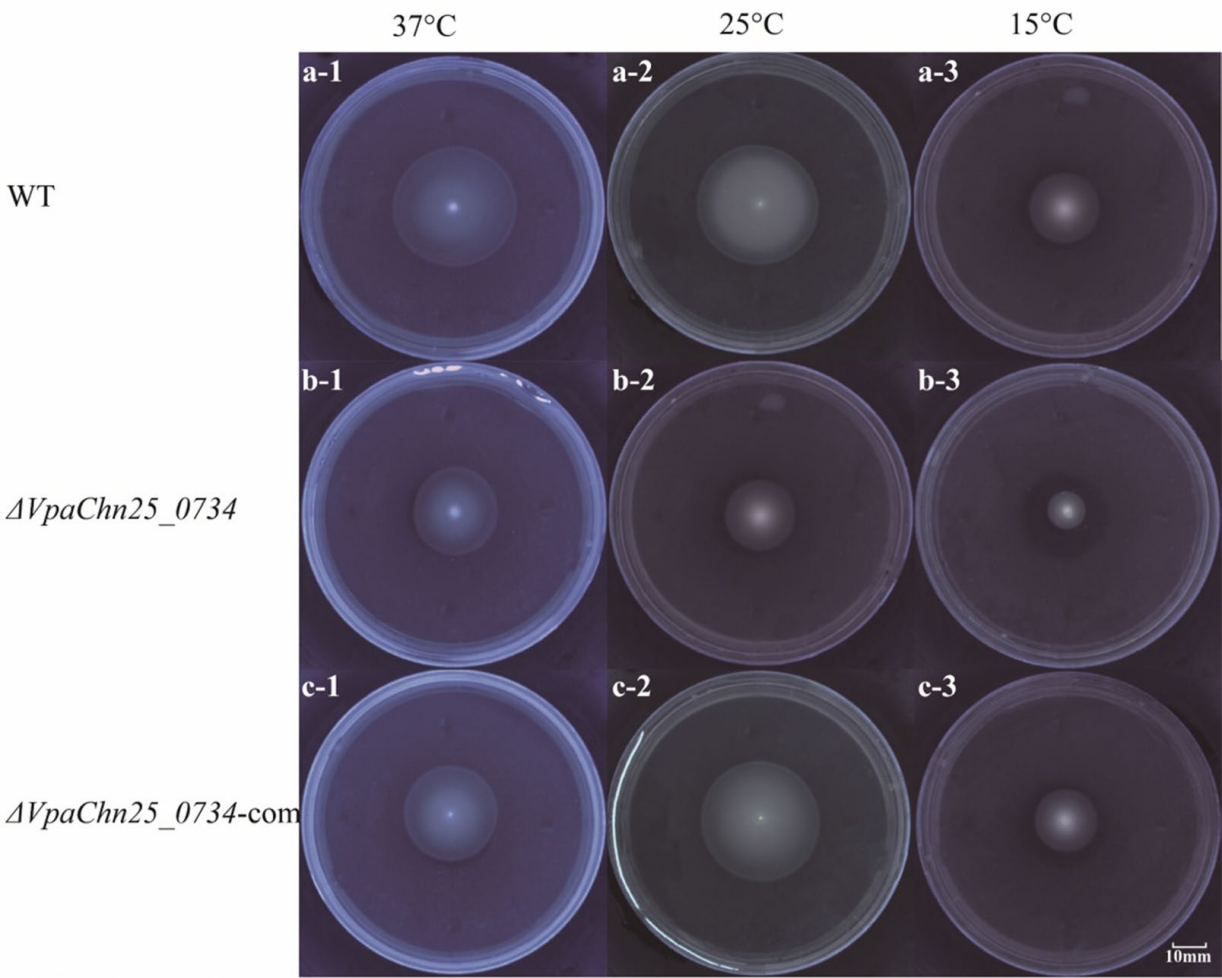
Swimming mobility of the WT, *ΔVpaChn25_0734* (543-bp), and *ΔVpaChn25_0734*-com (543-bp) strains at different temperatures. **a**–**c** 37 °C (**a-1**–**c-1**), 25 °C (**a-2**–**c-2**), and 15 °C (**a-3**–**c-3**), respectively

*Vibrio parahaemolyticus* is found to grow as swimming cells in liquid environments (Freitas et al. 2020). In this study, swimming mobility of the WT, *ΔVpaChn25_0734* (543-bp), and *ΔVpaChn25_0734*-com (543-bp) strains were examined at different temperatures. As shown in Fig. 3a-1 to c-1, no significant difference in swimming circles was observed among the three strains grown in semi-solid TSB agar medium (pH 8.5, 3% NaCl, 0.25% agar) at 37 °C (*p* > 0.05). However, at 25 °C, the *ΔVpaChn25_0734* (543-bp) mutant swam significantly slower (8.00 ± 0.71 mm) than the WT (12.75 ± 2.86 mm), and *ΔVpaChn25_0734*-com (543-bp) (9.00 ± 1.2 mm) strains, respectively (*p* < 0.05) (Fig. 3a-2 to c-2). The similar case was observed at 15 °C (Fig. 3a-3 to c-3). These results indicated that the deletion of *VpaChn25_0734* (543-bp) gene significantly inhibited swimming mobility of *V. parahaemolyticus* CHN25 at the lower temperatures.

### Biofilm formation of the *ΔVpaChn25_0734* (543-bp) mutant

**Fig. 4:**
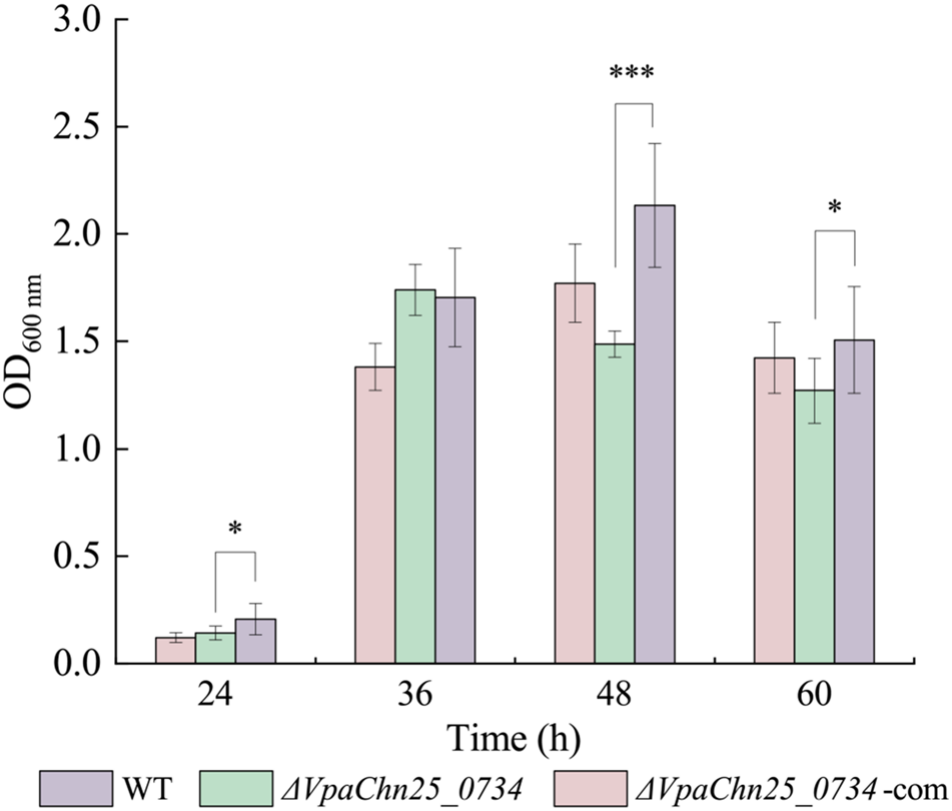
Biofilm formation of the WT, *ΔVpaChn25_0734* (543-bp), and *ΔVpaChn25_0734*-com (543-bp) strains in the TSB medium at 37 °C. *p < 0.05; ***p < 0.001 compared with the WT strain

Given the growth inhibition at the lower temperatures, the dynamic process of biofilm formation of the three strains was examined at 37 °C for 60 h using the crystalline violet staining method. All three strains established biofilms at three different stages (development, maturation, and diffusion) when grown in TSB medium (pH 8.5, 3% NaCl) in static culture condition, which was consistent with the previous report (Yang et al. 2020). Nevertheless, the biofilm biomass formed by the *ΔVpaChn25_0734* (543-bp) mutant was significantly less than the WT strain at development (24 h), maturation (48 h), and diffusion (60 h) stages (*p* < 0.05) (Fig. 4).

### Cell membrane permeability, hydrophobicity, and fluidity of the *ΔVpaChn25_0734* (543-bp) mutant

**Fig. 5:**
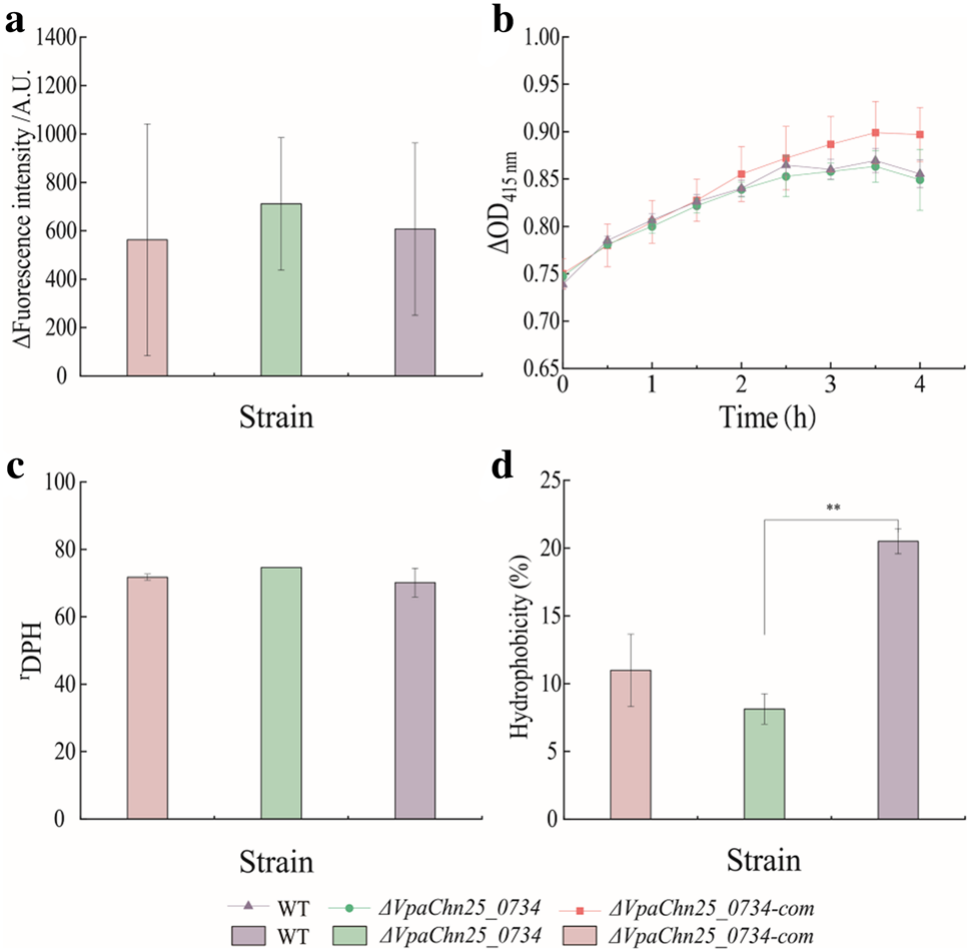
Cell membrane permeability and fluidity, and surface hydrophobicity of the WT, *ΔVpaChn25_0734* (543-bp), and *ΔVpaChn25_0734*-com (543-bp) strains. **a**–**d** Outer and internal membrane permeability (**a**, **b**), fluidity (**c**), and hydrophobicity (**d**). DPH: 1,6-diphenyl-1,3,5-hexatriene. **p < 0.01 compared with the WT strain

To investigate the impact of *VpaChn25_0734* (543-bp) gene deletion on cell structure of *V. parahaemolyticus* CHN25, we further examined cell membrane permeability and fluidity, and surface hydrophobicity of the three strains. As shown in Fig. 5, no significant difference in membrane permeability and fluidity was observed among the three strains grown in TSB medium (pH 8.5, 3% NaCl) at 37 °C (*p* > 0.05) (Fig. 5a–c). However, the cell surface hydrophobicity of the *ΔVpaChn25_0734* (543-bp) mutant was significantly decreased (2.52-fold) when compared to the WT (*p* < 0.01) (Fig. 5d).

### Secretome of the *ΔVpaChn25_0734* (543-bp) mutant

**Fig. 6:**
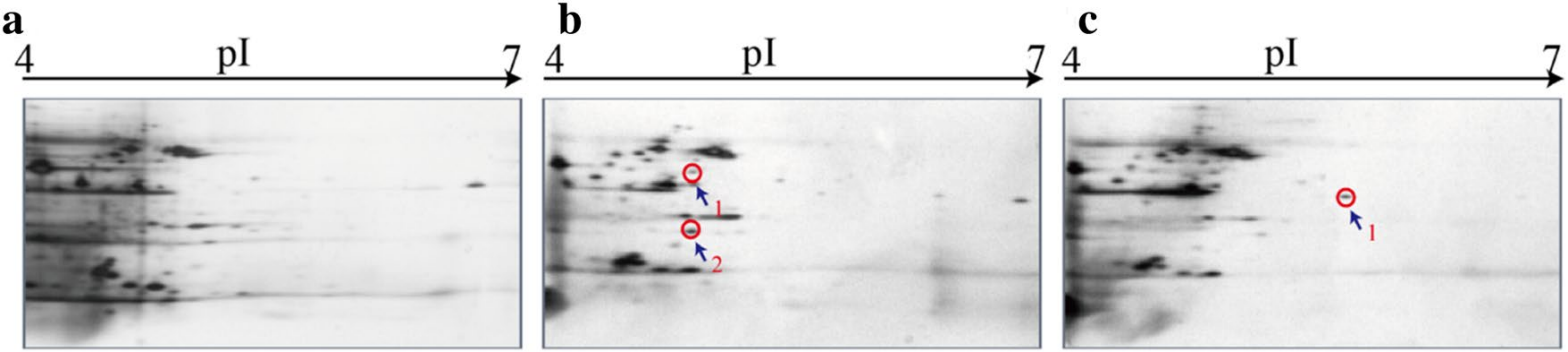
The 2D-GE analysis of extracellular proteins of the WT, *ΔVpaChn25_0734* (543-bp), and *ΔVpaChn25_0734*-com (543-bp) strains. **a**–**c** WT (**a**); *ΔVpaChn25_0734* (543-bp) (**b**); and *ΔVpaChn25_0734*-com (543-bp) (**c**), respectively

The *ΔVpaChn25_0734* (543-bp) mutant also grew more slowly than the WT strain when cultured in the TSB medium (pH 8.5, 3% NaCl) in static culture condition at 37 °C (data not shown), consistent with the results of the biofilm formation experiments. As shown in Fig. 6, slightly changed secretome profiles among the three strains were observed, based on the 2D-GE analysis. Three independent parallel 2D-GE gels of each sample produced identical results (data not shown). The different protein spots were excised from the 2D-GE gels and further identified by LC–MS/MS analysis.

As shown in Fig. 6, two differential extracellular proteins secreted by the *ΔVpaChn25_0734* (543-bp) mutant were identified: spot 34-b-1 was identified as a 30S ribosomal protein S1 (*RpsA*); and Spot 34-b-2 was identified as a DNA-directed RNA polymerase subunit alpha (*RpoA*) (Table 2).

**Table 2:** Identification of the differential protein spots on the secretome profiles by LC–MS/MS analysis

Protein spot	Uniprot no.	Protein	Gene	MW (Da)	pI	Score	Sequence coverage (%)
34-b-1	A0A0D1GGT4	30S ribosomal protein S1	*rpsA*	61,378.44	4.8	69.24	6.95
34-b-2	S5IPP6	DNA-directed RNA polymerase subunit alpha	*rpoA*	36,472.05	4.78	76.34	35.15
34-c-1	S5IV75	Aspartokinase	*M634_16115*	48,807.97	4.98	60.18	4.89

### Interaction between the *ΔVpaChn25_0734* (543-bp) mutant and host intestinal epithelial cells

**Fig. 7:**
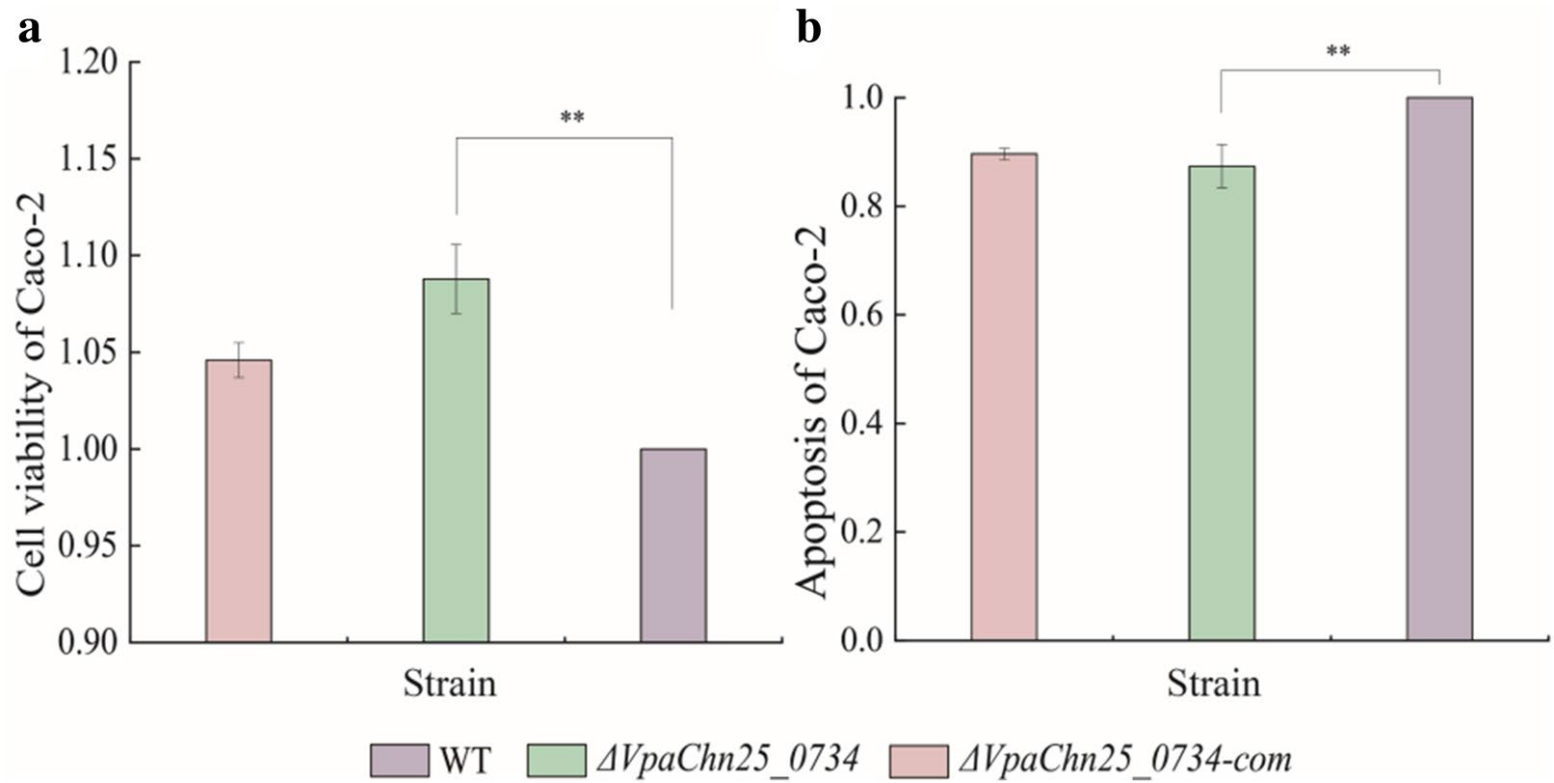
The viability and apoptosis of Caco-2 cells infected by the WT, *ΔVpaChn25_0734* (543-bp), and *ΔVpaChn25_0734*-com (543-bp) strains. (**a**) Cell viability; and (**b**) cell apoptosis. **p < 0.01 compared with the WT

As illustrated in Fig. 7, when compared to the infection with the WT strain, the viability of Caco-2 cells significantly increased 1.09-fold after infected with the *ΔVpaChn25_0734* (543-bp) mutant at 37 °C for 4 h (*p* < 0.01) (Fig. 7a). Meanwhile, Caco-2 cells were double-stained with membrane-linked protein V-FITC and PI, and analyzed by flow cytometry. The results showed that the *ΔVpaChn25_0734* (543-bp) mutant induced apoptosis of Caco-2 cells at a significantly reduced rate (0.87-fold) than the WT strain after the infection for 4 h (*p* < 0.01) (Fig. 7b). These results indicated a decrease in cytotoxicity of *V. parahaemolyticus* CHN25 in the absence of the *VpaChn25_0734* (543-bp) gene.

### Altered transcriptome of the *ΔVpaChn25_0734* (543-bp) mutant

**Fig. 8:**
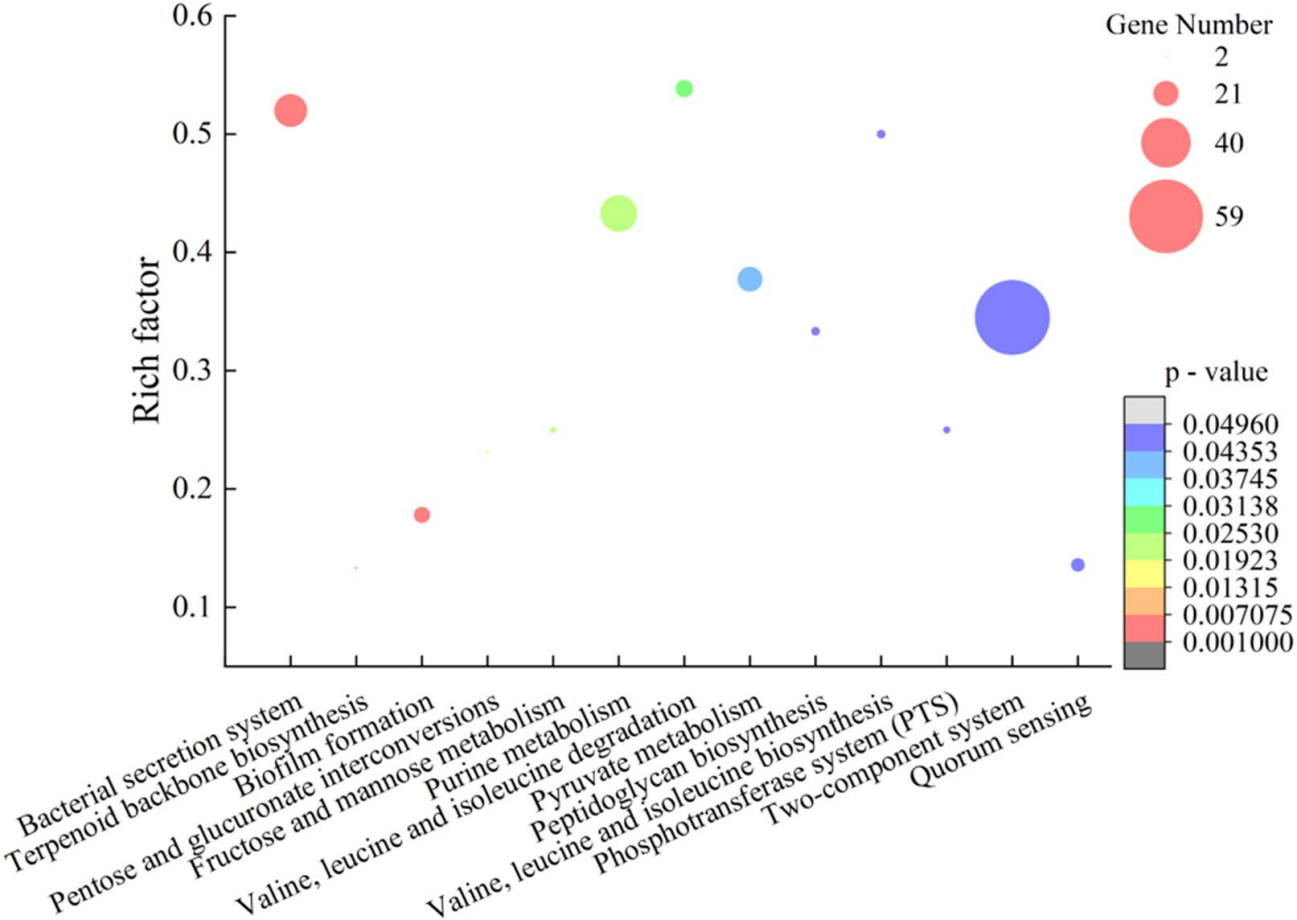
Thirteen metabolic pathways significantly altered in the *ΔVpaChn25_0734* (543-bp) mutant

To investigate global-level gene expression change mediated by the *VpaChn25_0734* (543-bp) gene deletion, transcriptomes of the WT, *ΔVpaChn25_0734* (543-bp), and *ΔVpaChn25_0734*-com (543-bp) strains were determined, when incubated in TSB medium (pH 8.5, 3% NaCl) at 37 °C. Comparative transcriptome analysis showed that approximately 17.03% of the genes in the *ΔVpaChn25_0734* (543-bp) mutant were differentially expressed, which were classified into various gene functional catalogues (Fig. 8). A complete list of the DEGs in the three strains was deposited in the NCBI SRA database (http://www.ncbi.nlm.nih.gov/sra/) under the accession number PRJNA733855. Expression of representative DEGs in the *ΔVpaChn25_0734* (543-bp) mutant were examined by the RT-PCR assay, and the resulting data were consistent with the transcriptomic analysis (Table S1).

### The major changed metabolic pathways in the *ΔVpaChn25_0734* (543-bp) mutant

Approximately thirteen significantly changed metabolic pathways were identified in the *VpaChn25_0734* (543-bp) mutant, including the bacterial secretion system; terpenoid backbone biosynthesis; biofilm formation; pentose and glucuronate interconversions; fructose and mannose metabolism; purine metabolism; valine, leucine, and isoleucine degradation; pyruvate metabolism; peptidoglycan biosynthesis; valine, leucine and isoleucine biosynthesis, phosphotransferase system (PTS), two-component system (T-CS), and quorum sensing (QS) (Fig. 8).

Remarkably, approximately 81 DEGs involved in 10 of the 13 metabolic pathways (except the terpenoid backbone biosynthesis, purine metabolism, and T-CS) were all significantly down-regulated in the *ΔVpaChn25_0734* (543-bp) mutant (0.051- to 0.500-fold) (*p* < 0.05), when compared with the WT and *ΔVpaChn25_0734-*com (543-bp) strains (Table 3).

**Table 3:** Major altered metabolic pathways in the *ΔVpaChn25_0734* (543-bp) mutant

Metabolic pathway	Gene ID	Fold change	Gene description
Bacterial secretion system	*VpaChn25_RS18155*	0.188	Protein translocase subunit SecF
	*VpaChn25_RS00645*	0.197	T2SS minor pseudopilin GspH
	*VpaChn25_RS13790*	0.214	T2SS/T6SS protein
	*VpaChn25_RS00650*	0.221	T2SS minor pseudopilin GspI
	*VpaChn25_RS08830*	0.223	T3SS needle filament protein VscF
	*VpaChn25_RS00640*	0.266	T2SS major pseudopilin GspG
	*VpaChn25_RS13760*	0.287	T2SS protein
	*VpaChn25_RS18150*	0.303	Protein translocase subunit SecD
	*VpaChn25_RS13775*	0.318	T2SS protein
	*VpaChn25_RS00670*	0.333	T2SS protein M
	*VpaChn25_RS00660*	0.335	T2SS minor pseudopilin GspK
	*VpaChn25_RS00655*	0.353	T2SS minor pseudopilin GspJ
	*VpaChn25_RS13765*	0.360	T2SS protein
	*VpaChn25_RS02885*	0.365	Preprotein translocase subunit YajC
	*VpaChn25_RS00630*	0.392	T2SS ATPase GspE
	*VpaChn25_RS00665*	0.413	T2SS protein GspL
	*VpaChn25_RS00635*	0.418	T2SS inner membrane protein GspF
	*VpaChn25_RS00625*	0.420	T2SS secretin GspD
	*VpaChn25_RS00470*	0.438	Sec-independent protein translocase subunit TatA
	*VpaChn25_RS02895*	0.500	Protein translocase subunit SecF
	*VpaChn25_RS20595*	2.166	T6SS protein TssL%2C long form
	*VpaChn25_RS08730*	2.534	SctR familyT3SS export apparatus subunit VscR
	*VpaChn25_RS20535*	2.837	T6SS ATPase TssH
	*VpaChn25_RS20605*	3.688	T6SS lipoprotein TssJ
	*VpaChn25_RS08715*	3.947	T3SS central stalk protein VscO
	*VpaChn25_RS02080*	4.037	Outer membrane channel protein TolC
Terpenoid backbone biosynthesis	*VpaChn25_RS01730*	2.392	Octaprenyl diphosphate synthase
	*VpaChn25_RS03685*	2.642	4-(cytidine 5'-diphospho)-2-C-methyl-D-erythritol kinase
Biofilm formation	*VpaChn25_RS22250*	0.051	Catalase
	*VpaChn25_RS01165*	0.346	UDP-N-acetyl-D-mannosamine dehydrogenase
	*VpaChn25_RS19800*	0.352	GGDEF and EAL domain-containing protein
	*VpaChn25_RS12510*	0.359	Porin
	*VpaChn25_RS12935*	0.378	Carbon storage regulator CsrA
	*VpaChn25_RS01180*	0.406	UDP-N-acetylglucosamine 2-epimerase (hydrolyzing)
	*VpaChn25_RS01160*	0.422	UDP-N-acetylglucosamine 2-epimerase (non-hydrolyzing)
	*VpaChn25_RS15210*	0.494	EAL domain-containing protein
	*VpaChn25_RS15250*	0.499	Class I adenylate cyclase
	*VpaChn25_RS01255*	2.169	UDP-N-acetylglucosamine 2-epimerase (non-hydrolyzing)
	*VpaChn25_RS18960*	2.719	Porin family protein
	*VpaChn25_RS04330*	3.800	Transmembrane regulator ToxS
	*VpaChn25_RS04335*	4.405	Transcriptional regulator ToxR
Pentose and glucuronate interconversions	*VpaChn25_RS23490*	0.423	L-arabinose isomerase
	*VpaChn25_RS16200*	0.462	Oligogalacturonate lyase family protein
	*VpaChn25_RS14015*	3.101	Ribulose-phosphate 3-epimerase
Fructose and mannose metabolism	*VpaChn25_RS01915*	0.132	Phosphoenolpyruvate-protein phosphotransferase
	*VpaChn25_RS21580*	0.222	Phospho-sugar mutase
	*VpaChn25_RS01930*	0.431	Mannitol-1-phosphate 5-dehydrogenase
	*VpaChn25_RS20215*	2.537	Mannose-6-phosphate isomerase%2C class I
	*VpaChn25_RS01350*	3.472	Class II fructose-bisphosphatase
Purine metabolism	*VpaChn25_RS03475*	0.165	LOG family protein
	*VpaChn25_RS11920*	0.172	Phosphopentomutase
	*VpaChn25_RS22525*	0.198	Purine-nucleoside phosphorylase
	*VpaChn25_RS11915*	0.252	Purine-nucleoside phosphorylase
	*VpaChn25_RS01570*	0.315	2'%2C3'-cyclic-nucleotide 2'-phosphodiesterase
	*VpaChn25_RS02805*	0.355	Exopolyphosphatase
	*VpaChn25_RS03725*	0.498	Bifunctional UDP-sugar hydrolase/5'-nucleotidase
	*VpaChn25_RS09575*	2.133	Ribonucleotide-diphosphate reductase subunit beta
	*VpaChn25_RS12730*	2.239	Hypoxanthine phosphoribosyltransferase
	*VpaChn25_RS16050*	2.239	Pyrimidine/purine nucleoside phosphorylase
	*VpaChn25_RS09525*	2.385	Ribonucleoside-diphosphate reductase subunit alpha
	*VpaChn25_RS03680*	2.618	Ribose-phosphate pyrophosphokinase
	*VpaChn25_RS19630*	2.870	Inosine/guanosine kinase
	*VpaChn25_RS17840*	3.332	Adenosine deaminase
	*VpaChn25_RS06885*	3.837	Formate-dependent phosphoribosylglycinamide formyltransferase
	*VpaChn25_RS06070*	4.237	Adenylosuccinate lyase
	*VpaChn25_RS03015*	4.441	Glutamine-hydrolyzing GMP synthase
	*VpaChn25_RS03010*	4.645	IMP dehydrogenase
	*VpaChn25_RS18400*	4.849	GMP reductase
	*VpaChn25_RS15545*	5.336	5-(Carboxyamino)imidazole ribonucleotide synthase
	*VpaChn25_RS01595*	5.550	Adenylyl-sulfate kinase
	*VpaChn25_RS03315*	5.727	Phosphoribosylformylglycinamidine synthase
	*VpaChn25_RS15540*	6.048	5-(Carboxyamino)imidazole ribonucleotide mutase
	*VpaChn25_RS14820*	6.718	Phosphoribosylamine-glycine ligase
	*VpaChn25_RS01585*	7.035	Sulfate adenylyltransferase subunit CysN
	*VpaChn25_RS06715*	7.054	Phosphoribosylaminoimidazolesuccinocarboxamide synthase
	*VpaChn25_RS01580*	7.268	Sulfate adenylyltransferase subunit CysD
	*VpaChn25_RS11200*	9.446	Phosphoribosylformylglycinamidine cyclo-ligase
	*VpaChn25_RS14815*	9.555	Bifunctional phosphoribosylaminoimidazolecarboxamide formyltransferase/IMP cyclohydrolase
Valine, leucine and isoleucine degradation	*VpaChn25_RS20950*	0.207	Enoyl-CoA hydratase/isomerase family protein
	*VpaChn25_RS20945*	0.240	Methylcrotonoyl-CoA carboxylase
	*VpaChn25_RS20905*	0.255	3-hydroxyisobutyrate dehydrogenase
	*VpaChn25_RS20955*	0.279	Hydroxymethylglutaryl-CoA lyase
	*VpaChn25_RS20940*	0.385	Isovaleryl-CoA dehydrogenase
	*VpaChn25_RS20925*	0.422	CoA-acylating methylmalonate-semialdehyde dehydrogenase
	*VpaChn25_RS22255*	0.473	NAD(P)-dependent oxidoreductase
	*VpaChn25_RS15655*	0.484	Branched-chain-amino-acid transaminase
	*VpaChn25_RS18590*	3.000	3-hydroxyisobutyrate dehydrogenase
	*VpaChn25_RS18535*	3.301	Hydroxymethylglutaryl-CoA lyase
	*VpaChn25_RS18545*	3.733	Enoyl-CoA hydratase/isomerase family protein
	*VpaChn25_RS18550*	4.207	Methylcrotonoyl-CoA carboxylase
	*VpaChn25_RS18555*	5.096	Isovaleryl-CoA dehydrogenase
	*VpaChn25_RS18570*	9.201	CoA-acylating methylmalonate-semialdehyde dehydrogenase
Pyruvate metabolism	*VpaChn25_RS10085*	0.140	Pyruvate kinase
	*VpaChn25_RS22640*	0.175	FMN-dependent L-lactate dehydrogenase LldD
	*VpaChn25_RS16545*	0.233	2-hydroxyacid dehydrogenase
	*VpaChn25_RS12875*	0.255	Sodium-extruding oxaloacetate decarboxylase subunit alpha
	*VpaChn25_RS10425*	0.320	Lactoylglutathione lyase
	*VpaChn25_RS06690*	0.352	NAD-dependent malic enzyme
	*VpaChn25_RS20420*	0.360	D-lactate dehydrogenase
	*VpaChn25_RS14690*	0.377	Class II fumarate hydratase
	*VpaChn25_RS12870*	0.387	Sodium ion-translocating decarboxylase subunit beta
	*VpaChn25_RS20930*	0.389	Thiolase family protein
	*VpaChn25_RS14715*	0.400	Acetate-CoA ligase
	*VpaChn25_RS12740*	0.410	Dihydrolipoyl dehydrogenase
	*VpaChn25_RS12745*	0.414	Pyruvate dehydrogenase complex dihydrolipoyllysine-residue acetyltransferase
	*VpaChn25_RS22970*	0.435	Formate C-acetyltransferase/glycerol dehydratase family glycyl radical enzyme
	*VpaChn25_RS17630*	0.488	Phosphoenolpyruvate synthase
	*VpaChn25_RS08880*	2.772	Aldehyde dehydrogenase
	*VpaChn25_RS02855*	5.609	Malate synthase A
	*VpaChn25_RS17335*	6.710	VOC family protein
	*VpaChn25_RS18565*	17.794	Thiolase family protein
	*VpaChn25_RS16830*	25.569	Malate synthase
Peptidoglycan biosynthesis	*VpaChn25_RS02225*	0.374	Phospho-N-acetylmuramoyl-pentapeptide-transferase
	*VpaChn25_RS02230*	0.381	UDP-N-acetylmuramoyl-L-alanine-D-glutamate ligase
	*VpaChn25_RS02220*	0.394	UDP-N-acetylmuramoyl-tripeptide-D-alanyl-D-alanine ligase
	*VpaChn25_RS14065*	0.471	PBP1A family penicillin-binding protein
	*VpaChn25_RS23100*	0.493	D-alanyl-D-alanine carboxypeptidase
	*VpaChn25_RS02245*	0.498	UDP-N-acetylmuramate-L-alanine ligase
	*VpaChn25_RS23215*	3.035	Monofunctional biosynthetic peptidoglycan transglycosylase
Valine, leucine and isoleucine biosynthesis	*VpaChn25_RS01810*	0.230	3-isopropylmalate dehydratase large subunit
	*VpaChn25_RS01820*	0.231	2-isopropylmalate synthase
	*VpaChn25_RS01815*	0.236	3-isopropylmalate dehydrogenase
	*VpaChn25_RS01805*	0.278	3-isopropylmalate dehydratase small subunit
	*VpaChn25_RS15665*	0.392	Threonine ammonia-lyase%2C biosynthetic
	*VpaChn25_RS15660*	0.402	Dihydroxy-acid dehydratase
	*VpaChn25_RS00150*	0.499	Ketol-acid reductoisomerase
Phosphotransferase system (PTS)	*VpaChn25_RS19520*	0.180	PTS fructose transporter subunit IIBC
	*VpaChn25_RS13650*	0.257	PTS IIA-like nitrogen regulatory protein PtsN
	*VpaChn25_RS13660*	0.322	HPr family phosphocarrier protein
	*VpaChn25_RS22265*	0.362	PTS fructose transporter subunit IIB
	*VpaChn25_RS22270*	0.429	PTS sugar transporter subunit IIA
	*VpaChn25_RS21735*	3.677	PTS sugar transporter subunit IIA
Two-component system	*VpaChn25_RS01005*	0.131	Methyl-accepting chemotaxis protein
	*VpaChn25_RS09025*	0.162	Response regulator
	*VpaChn25_RS21540*	0.221	COX15/CtaA family protein
	*VpaChn25_RS02375*	0.276	Aerobic respiration two-component sensor histidine kinase ArcB
	*VpaChn25_RS13640*	0.283	RNA polymerase factor sigma-54
	*VpaChn25_RS09390*	0.291	Response regulator transcription factor
	*VpaChn25_RS16750*	0.319	Sigma-54-dependent Fis family transcriptional regulator
	*VpaChn25_RS20290*	0.341	Anaerobic C4-dicarboxylate transporter
	*VpaChn25_RS02065*	0.350	Methyl-accepting chemotaxis protein
	*VpaChn25_RS18220*	0.352	Response regulator
	*VpaChn25_RS05680*	0.370	Cytochrome bd-I oxidase subunit CydX
	*VpaChn25_RS22605*	0.376	Methyl-accepting chemotaxis protein
	*VpaChn25_RS15910*	0.410	Methyl-accepting chemotaxis protein
	*VpaChn25_RS19275*	0.425	Response regulator
	*VpaChn25_RS09805*	0.429	Methyl-accepting chemotaxis protein
	*VpaChn25_RS23375*	0.436	Methyl-accepting chemotaxis protein
	*VpaChn25_RS06230*	0.442	Trimethylamine-N-oxide reductase TorA
	*VpaChn25_RS00235*	0.443	ABC transporter permease
	*VpaChn25_RS02795*	0.446	Phosphate regulon sensor histidine kinase PhoR
	*VpaChn25_RS20400*	0.472	Methyl-accepting chemotaxis protein
	*VpaChn25_RS11070*	0.498	Flagellin
	*VpaChn25_RS18435*	2.066	Branched-chain amino acid ABC transporter permease
	*VpaChn25_RS19580*	2.127	MFS transporter
	*VpaChn25_RS08625*	2.144	Tripartite tricarboxylate transporter substrate-binding protein
	*VpaChn25_RS21815*	2.171	Efflux RND transporter periplasmic adaptor subunit
	*VpaChn25_RS17530*	2.189	ABC transporter permease
	*VpaChn25_RS21290*	2.283	Methyl-accepting chemotaxis protein
	*VpaChn25_RS02145*	2.313	Ubiquinol-cytochrome c reductase iron-sulfur subunit
	*VpaChn25_RS04780*	2.316	TRAP transporter small permease
	*VpaChn25_RS20970*	2.373	PAS domain S-box protein
	*VpaChn25_RS18440*	2.391	Branched-chain amino acid ABC transporter permease
	*VpaChn25_RS23155*	2.442	Polysaccharide biosynthesis tyrosine autokinase
	*VpaChn25_RS07955*	2.487	Cytochrome-c oxidase%2C cbb3-type subunit I
	*VpaChn25_RS09955*	2.525	Polysulfide reductase NrfD
	*VpaChn25_RS18255*	2.568	Methyl-accepting chemotaxis protein
	*VpaChn25_RS22465*	2.662	Methyl-accepting chemotaxis protein
	*VpaChn25_RS12590*	2.713	ABC transporter ATP-binding protein
	*VpaChn25_RS05585*	2.742	Two-component system response regulator TorR
	*VpaChn25_RS21255*	2.753	Phosphate ABC transporter substrate-binding protein
	*VpaChn25_RS07030*	3.314	Polyamine ABC transporter substrate-binding protein
	*VpaChn25_RS23145*	3.337	Polysaccharide export protein
	*VpaChn25_RS23150*	3.370	Protein-tyrosine-phosphatase
	*VpaChn25_RS10335*	3.461	Peptide ABC transporter substrate-binding protein
	*VpaChn25_RS10315*	3.653	Murein tripeptide/oligopeptide ABC transporter ATP-binding protein OppF
	*VpaChn25_RS10320*	3.922	ABC transporter ATP-binding protein
	*VpaChn25_RS08635*	3.799	Tripartite tricarboxylate transporter permease
	*VpaChn25_RS07090*	4.041	Oligopeptide ABC transporter permease OppB
	*VpaChn25_RS12585*	4.130	ABC transporter ATP-binding protein
	*VpaChn25_RS20195*	4.309	Hexose-6-phosphate: phosphate antiporter
	*VpaChn25_RS10325*	5.064	Oligopeptide ABC transporter permease OppC
	*VpaChn25_RS12575*	5.102	ABC transporter permease
	*VpaChn25_RS07085*	5.558	ABC transporter permease subunit
	*VpaChn25_RS12580*	5.629	ABC transporter permease
	*VpaChn25_RS10330*	5.675	Oligopeptide ABC transporter permease OppB
	*VpaChn25_RS00555*	12.578	Nitrogen regulation protein NR(II)
	*VpaChn25_RS18425*	14.069	ABC transporter ATP-binding protein
	*VpaChn25_RS00550*	14.169	Nitrogen regulation protein NR(I)
	*VpaChn25_RS12570*	15.367	ABC transporter substrate-binding protein
	*VpaChn25_RS00565*	15.620	Glutamate-ammonia ligase
Quorum sensing	*VpaChn25_RS09030*	0.103	Sensor histidine kinase N-terminal domain-containing protein
	*VpaChn25_RS12840*	0.354	S-ribosylhomocysteine lyase
	*VpaChn25_RS18275*	0.418	3-deoxy-7-phosphoheptulonate synthase AroG
	*VpaChn25_RS25650*	0.427	GTP cyclohydrolase II
	*VpaChn25_RS09700*	0.447	Aminodeoxychorismate/anthranilate synthase component II
	*VpaChn25_RS09695*	0.485	Anthranilate synthase component 1
	*VpaChn25_RS01845*	2.207	Long-chain fatty acid-CoA ligase
	*VpaChn25_RS07080*	2.757	ATP-binding cassette domain-containing protein
	*VpaChn25_RS12735*	2.898	Transcriptional regulator OpaR
	*VpaChn25_RS18430*	3.288	Long-chain fatty acid-CoA ligase
	*VpaChn25_RS07075*	3.456	ATP-binding cassette domain-containing protein

In the pentose and glucuronate interconversions, expression of two DEGs were significantly repressed at the transcriptional level (0.423-, and 0.462-fold) (*p* < 0.05), which encoded an l-arabinose isomerase (*VpaChn25_RS23490*), and an oligogalacturonate lyase family protein (*VpaChn25_RS16200*), respectively. In the fructose and mannose metabolism, 3 DEGs encoding key enzymes were also significantly inhibited (0.132- to 0.431-fold) (*p* < 0.05), including a phosphoenolpyruvate-protein phosphotransferase (*VpaChn25_RS01915*), a phospho-sugar mutase (*VpaChn25_RS21580*), and a mannitol-1-phosphate 5-dehydrogenase (*VpaChn25_RS01930*). In the pyruvate metabolism, expression of 15 DGEs, e.g., a key pyruvate kinase (*VpaChn25_RS10085*) and a 2-hydroxyacid dehydrogenase (*VpaChn25_RS16545*), were significantly depressed (0.140- to 0.488-fold). Additionally, 21 DGEs involved in T-CS were significantly down-regulated (0.131- to 0.498-fold). All these metabolic pathways were involved in carbohydrate metabolism, and the overall down-regulation trend of the DGEs sets suggested under-activity of carbon source transport and/or utilization, which may have consequently led to a certain impairment of energy supply.

In the peptidoglycan biosynthesis, expression of 6 DGEs were significantly down-regulated (0.374- to 0.498-fold), including a phospho-*N*-acetylmuramoyl-pentapeptide-transferase (*VpaChn25_RS02225*), a UDP-*N*-acetylmuramoyl-l-alanine-d-glutamate ligase (*VpaChn25_RS02230*), a UDP-*N*-acetylmuramoyl-tripeptide-d-alanyl-d-alanine ligase (*VpaChn25_RS02220*), a PBP1A family penicillin-binding protein (*VpaChn25_RS14065*), a d-alanyl-d-alanine carboxypeptidase (*VpaChn25_RS23100*), and a UDP-*N*-acetylmuramate-l-alanine ligase (*VpaChn25_RS02245*). Inhibition of the peptidoglycan biosynthetic may have compromised cell integrity and inhibited the normal growth and division of the *ΔVpaChn25_0734* (543-bp) mutant.

In bacterial secretion systems, remarkably, all 13 DEGs involved in type II secretion system (T2SS) were significantly down-regulated (0.197- to 0.420-fold) (*p* < 0.05), including T2SS minor pseudopilins GspH, GspI, GspG, GspK, and GspJ (*VpaChn25_RS00645*, *VpaChn25_RS00650*, *VpaChn25_RS00640*, *VpaChn25_RS00660*, and *VpaChn25_RS00655*); T2SS proteins M (*VpaChn25_RS00670*) and GspL (*VpaChn25_ RS00665*); T2SS ATPase GspE (*VpaChn25_RS00630*); T2SS inner membrane protein GspF (*VpaChn25_RS00635*); T2SS secretin GspD (*VpaChn25_RS00625*); and T2SS proteins (*Vpachn25_RS13760*, *Vpachn25_RS13775*, and *Vpachn25_RS13765*). Unexpectedly, expression of 3 DGEs involved in type VI secretion system (T6SS) were enhanced (2.166- to 3.688-fold) (*p* < 0.05), including T6SS protein TssL (*VpaChn25_RS20595*), ATPase TssH (*VpaChn25_RS20535*), and lipoprotein TssJ (*VpaChn25_RS20605*). T6SS secrets bacterial toxins and regulates biofilm formation or stress sensing (Aschtgen et al. 2010). It was speculated that the depressed T2SS induced by the deletion of *VpaChn25_0734* (543-bp) may have triggered T6SS for more support in material transport and nutrient uptake.

**Fig. 9:**
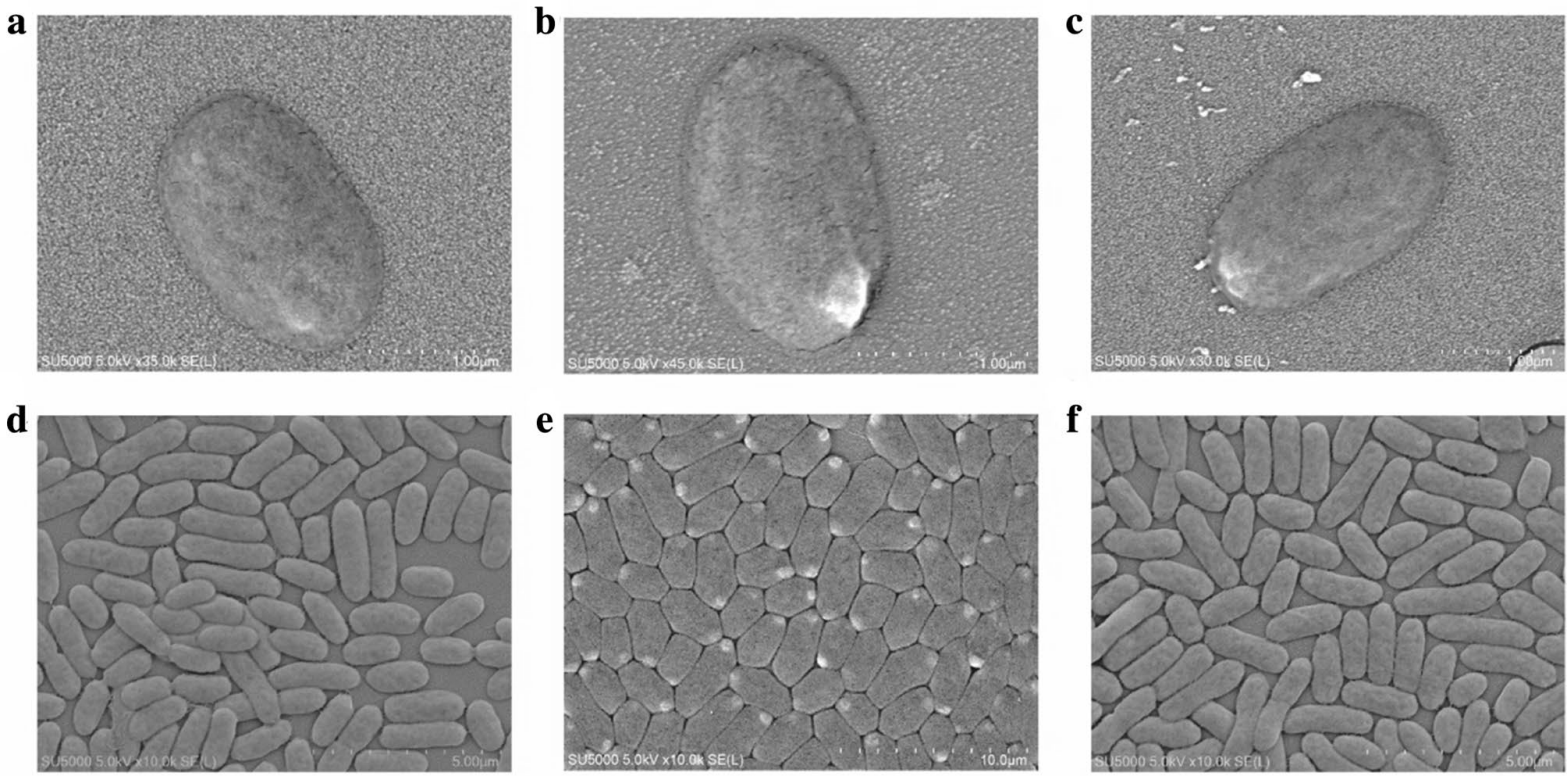
The SEM observation of cell structure of the WT, *ΔVpaChn25_0734* (543-bp), and *ΔVpaChn25_0734*-com (543-bp) strains. **a**, **d** WT; **b**, **e** *ΔVpaChn25_0734*; and **c**, **f** *ΔVpaChn25_0734*-com

In the biofilm formation, expression of 9 DGEs, e.g., a catalase (*VpaChn25_RS22250*), a porin (*VpaChn25_RS12510*), and a carbon storage regulator CsrA (*VpaChn25_RS12935*), was significantly depressed (0.051- to 0.499-fold) (*p* < 0.05). Additionally, 6 DGEs involved in the QS (0.103- to 0.485-fold) also showed a decrease in transcriptional levels in the *ΔVpaChn25_0734* (543-bp) mutant. The SEM observation of the three strains provided cell structure evidence for the above results, in which more cytocysts were obviously present in the *ΔVpaChn25_0734* (543-bp) mutant than the WT and *ΔVpaChn25_0734*-com (543-bp) strains (Fig. 9).

Comparative transcriptomic analysis also revealed a few metabolic pathways that were significantly up-regulated in the *ΔVpaChn25_0734* (543-bp) mutant (*p* < 0.05), such as purine metabolism, and terpenoid backbone biosynthesis. Moreover, there were several DEGs with greatly changed transcription levels (12.578- to 25.569-fold) mediated by the *VpaChn25_0734* (543-bp) gene deletion. For example, expression of 22 DEGs in the purine metabolism was significantly up-regulated (2.133- to 9.555-fold) (*p* < 0.05). Additionally, a highly up-regulated gene *VpaChn25_RS18565* (17.794-fold) coded for a thiolase that catalyzes the reverse Claisen condensation reaction, the first step of the biosynthesis of sterols and ketone bodies in lipid metabolism (Anbazhagan et al. 2014). The gene (*VpaChn25_RS16830*, 25.569-fold) encoding a malate synthase, a key enzyme responsible for malic acid synthesis in the glyoxylate cycle (Pua et al. 2003), was also greatly up-regulated. Taken, these DEGs elicited by the deletion of *VpaChn25_0734* (543-bp) likely impacted multiple metabolic pathways and consequently resulted in the defective phenotypes of the *ΔVpaChn25_0734* (543-bp) mutant.

### Distribution of the *VpaChn25_0734* (543-bp) gene in bacteria

**Fig. 10:**
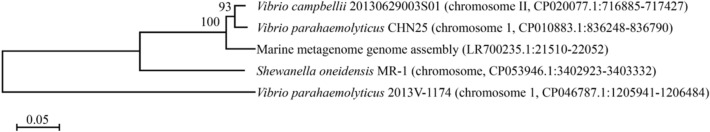
Phylogenetic relationships between the *VpaChn25_0734* (543-bp) gene and its homologues

A total of 119 *V. parahaemolyticus* isolates from aquatic products collected in Shanghai, China (Su and Chen 2020) were tested for the *VpaChn25_0734* (543-bp) gene by the PCR assay. The results revealed that approximately 0.84% (*n* = 1) of the *V. parahaemolyticus* isolates carried the *VpaChn25_0734* (543-bp) homologue. Moreover, sequence analysis against the GenBank database showed that *VpaChn25_0734* (543-bp) homologues were present in one *Vibrio* phage (vB_VpaM_VP-3212) (marine metagenome genome assembly, Genbank accession no.: LR700235); two *Vibrio* species, including *Vibrio campbellii* (Genbank accession no.: CP020077) and *V. parahaemolyticus* (Genbank accession no.: CP046787); and non-*Vibrio* genus *Shewanella oneidensis* (Genbank accession no.: CP053946). Based on these identified homologues, a phylogenetic tree was constructed (Fig. 10). This analysis revealed that the *VpaChn25_0734* (543-bp) gene was phylogenetically close to a *Vc3S01_A0696* gene of *V. campbellii* 2013 0629003S1. *V. campbellii* is an emerging aquaculture pathogen that causes Vibriosis in farmed shrimp (Nuidate et al. 2021). These results indicated that the *VpaChn25_0734* (543-bp) gene exists in *V. parahaemolyticus* population, within the *Vibrio* genus, and even across bacterial genera.

## Discussion

*Vibrio parahaemolyticus* is frequently isolated from seafoods at water temperatures above 15 °C (Su and Chen 2020). Huge amounts of phages in aquatic ecosystems allow genetic material to jump between strains and species (Lerminiaux and Cameron 2019). Currently, biological function of prophage-encoded genes in *V. parahaemolyticus* is not yet fully understood. In this study, one such gene *VpaChn25_0734* (543-bp) encoding a predicted phage morphogenetic protein in *V. parahaemolyticus* CHN25 genome was for the first time subjected to a systematic study. We successfully constructed the *ΔVpaChn25_0734* (543-bp) mutant and obtained its revertant *ΔVpaChn25_0734-*com (543-bp). Our data indicated that the deletion of *VpaChn25_0734* (543-bp) not only resulted in the growth hindrance of *V. parahaemolyticus* CHN25, particularly at lower temperatures, but also reduced the bacterial tolerance at pH 7.0–8.5. Flagella play a crucial role in bacterial pathogenesis linked to colonization, chemotaxis, biofilm formation, and virulence (Chevance and Hughes 2008; Erhardt 2016). Flagellar motility is one of the high-energy–utilizing cellular processes (Khan et al. 2020). In this study, our results indicated that the swimming mobility of *ΔVpaChn25_0734* (543-bp) was significantly inhibited at lower temperatures. Moreover, the *ΔVpaChn25_0734* (543-bp) mutant had a defect to a certain extent in all three stages of biofilm formation. These results indicated that the prophage-encoded *VpaChn25_0734* (543-bp) gene amplified the environmental persistence of *V. parahaemolyticus* CHN25.

Bacteria have evolved several types of secretion systems to secrete relevant substrates and effectors for bacterial adaptation to the environment and interaction with hosts (Costa et al. 2015; Wang et al. 2021b). In this study, comparative secretome analysis revealed a few differentially extracellular proteins secreted by the *ΔVpaChn25_0734* (543-bp) mutant. For example, Spot 34-b-1 was identified as a 30S ribosomal protein S1, which is mainly involved in protein synthesis in bacteria. Despite being present intracellularly, however, it was also detected in secreted proteins of *Streptococcus suis* and *Haemophilus parasuis* (Wu et al. 2008; Wei et al. 2014). Spot 34-b-2 was a DNA-directed RNA polymerase subunit alpha protein, and its dimerization is the first step in the sequential assembly of subunits to form the holoenzyme (Degen et al. 2014). Additionally, another protein Spot 34-c-1 was identified as an aspartate kinase in the biosynthesis of the aspartate family of amino acids including methionine, threonine, and lysine (Zou et al. 2020). *V. parahaemolyticus* infection resulted in autophagy, cell rounding, and cell lysis, which led to proinflammatory death of infected host cells (Shimohata et al. 2011). In this study, the in vitro cell model analysis revealed that the viability of Caco-2 cells was significantly increased after the *ΔVpaChn25_0734* (543-bp) mutant infected at 37 °C for 4 h, when compared with the WT strain (*p* < 0.05). Moreover, *ΔVpaChn25_0734* induced the apoptosis of Caco-2 cells at a lower rate than WT. Taken, the differentially secreted proteins by *ΔVpaChn25_0734* and or the expression of *VpaChn25_0734* (543-bp) gene benefited to *V. parahaemolyticus* CHN25 for its infection to the host cells.

Comparative transcriptome analysis revealed 13 significantly changed metabolic pathways in the *VpaChn25_0734* (543-bp) mutant. Remarkably, all the DEGs associated with the T2SS were significantly inhibited (0.197- to 0.42-fold) (*p* < 0.05), including GspH, GspI, GspG, GspK, GspJ, GspL, GspE, GspF, GspD, *Vpachn25_RS00670*, *Vpachn25_RS13760*, *Vpachn25_RS13775*, *Vpachn25_RS13765*. The main function of bacterial T2SS is to obtain nutrients via exoproteins such as hydrolases to degrade biopolymers. T2SS also promote the secretion of toxins, adhesins, mucin, or cytochromes involved in respiration, motility, or biofilm formation (Nivaskumar and Francetic 2014; Yu et al. 2015; Matsuda et al. 2019). For example, in this study, expression of T2SS minor pseudopilin GspK (*VpaChn25_RS00660*) was significantly decreased (0.335-fold) in the *ΔVpaChn25_0734* mutant. In T2SS, GspK binds on GspE N-domain via GspL and promotes GspE binding to cardiolipin to stimulate the initial rounds of ATP hydrolysis, which is required for assembly of GspG (Camberg et al. 2007; Cisneros et al. 2012). In this study, expression of GspH (*Vpachn25_RS00645*) was also highly repressed (0.197-fold). In T2SS, GspH binds the initiating tip complex in vitro via its globular domain, which could ensure a transition between initiation and ATPase-catalyzed elongation required for maximal efficiency of protein secretion under native conditions (Douzi et al. 2009; Korotkov and Sandkvist 2019). Similarly, three DEGs involved in the secretory-signal recognition particle (Sec-SRP) system were significantly down-regulated (0.188- to 0.5-fold) (*p* < 0.05), including a protein translocase subunit SecF (*VpaChn25_RS18155*), a protein translocase subunit SecD (*VpaChn25_RS18150*), and a protein translocase subunit SecF (*VpaChn25_RS02895*). These results suggested inactive nutrient uptake and/or biofilm formation of the *ΔVpaChn25_0734* (543-bp) mutant. Moreover, the DEG (*VpaChn25_RS22250*) encoding a catalase in the biofilm system was greatly down-regulated (0.051-fold). Catalase prevents the accumulation of hydrogen peroxide and protects cellular organelles from damage by peroxide (Goyal and Basak 2010). Additionally, the DEG (*VpaChn25_RS12510*) in the porin synthesis, which is a part of the biofilm system, was also decreased (0.359-fold). Porins are non-specific protein channels in bacterial outer membrane that enable the influx of hydrophilic solutes (Ades 2004; Liu et al. 2012). It will be interesting to further investigate cell location of the *VpaChn25_0734* (543-bp)-encoding protein in *V. parahaemolyticus* CHN25 in the future research.

The PTS functions in carbohydrate transport and regulates numerous cellular processes (Schauder et al. 1998; Galinier and Deutscher 2017). In this study, comparative transcriptome data revealed that the *VpaChn25_RS19520*, *VpaChn25_RS13650*, and *VpaChn25_RS22265* genes in PTS were significantly down-regulated (0.18, 0.257, 0.362-fold) in the *VpaChn25_0734* (543-bp) mutant. Meanwhile, 20 DEGs involved in carbohydrate metabolism also showed significantly down-regulated transcription (0.132- to 0.488-fold). For example, the DEG encoding a pyruvate kinase (PK, *VpaChn25_RS10085)* in the pyruvate metabolism was highly down-regulated (0.140-fold). PK catalyzes the last step of glycolysis, in which phosphoenolpyruvate is converted to pyruvate with the production of adenosine triphosphate (ATP) (Israelsen and Vander Heiden 2015). Additionally, 21 DGEs involved in T-CS were also significantly down-regulated (0.131- to 0.498-fold). ATP-dependent ABC transporters actively transport molecules across the lipid membrane (Moussatova et al. 2008). The overall down-regulation trend of the DGEs sets in carbohydrate metabolism suggested that the deletion of *VpaChn25_0734* (543-bp) gene negatively impacted nutrient acquisition and utilization, and consequently affected energy production and consumption of *V. parahaemolyticus* CHN25, consistent with defective phenotypes observed in this study.

Additionally, some DGEs were highly up-regulated in the *VpaChn25_0734* (543-bp) mutant. For example, expression of an inosine monophosphate (IMP) dehydrogenase (*VpaChn25_RS03010*) was increased by 4.645-fold. IMP dehydrogenase is the key enzyme in the de novo biosynthesis of guanosine monophosphate (GMP) (Salomon et al. 2004). In this study, expression of an octaprenyl diphosphate synthase (*ispB*, *VpaChn25_RS01730*) in the terpenoid backbone biosynthesis was increased by 2.392-fold, which was essential for the growth of *E. coli* (Okada et al. 1997; Helfrich et al. 2019). Additionally, the DEG encoding a 4-(cytidine 5′-diphospho)-2-C-methyl-d-erythritol kinase (*VpaChn25_RS03685*, 2.642-fold) was also significantly up-regulated. This enzyme is essential for isoprenoid biosynthesis in some pathogenic microorganisms (Wada et al. 2003). The interaction between *VpaChn25_0734* (543-bp) and these DEGs should be further investigated in future research.

## Conclusions

In the present study, we characterized for the first time the prophage-encoded gene *VpaChn25_0734* (543-bp) in *V. parahaemolyticus* CHN25 genome. The *ΔVpaChn25_0734* (543-bp) mutant was obtained and the revertant *ΔVpaChn25_0734*-com (543-bp) was also constructed. The *ΔVpaChn25_0734* (543-bp) mutant was defective in growth and swimming mobility particularly at lower temperatures, as well as in tolerance at pH 7.0–8.5. In the absence of the *VpaChn25_0734* (543-bp) gene, cell surface hydrophobicity and biofilm formation at all stages of *V. parahaemolyticus* CHN25 were significantly decreased (*p* < 0.05). These results indicated that the prophage-encoded *VpaChn25_0734* (543-bp) gene amplified environmental persistence of *V. parahaemolyticus* CHN25.

Comparative secretomic analysis revealed a slightly increased extracellular proteins secreted by the *ΔVpaChn25_0734* (543-bp) mutant, when compared with the WT and *ΔVpaChn25_0734*-com (543-bp) strains. Based on the Caco-2 cell model in vitro, the deletion of *VpaChn25_0734* (543-bp) gene significantly reduced the cytotoxicity of *V. parahaemolyticus* CHN25 to human intestinal epithelial cells (*p* < 0.05), which indicated that the *VpaChn25_0734* (543-bp) gene enhanced *V. parahaemolyticus* CHN25 fitness for surviving in the host.

Comparative transcriptome analysis revealed 13 significantly changed metabolic pathways in the *ΔVpaChn25_0734* (543-bp) mutant, showing significantly down-regulated carbon source transport and/or utilization, biofilm formation, and T2SS (*p* < 0.05), consistent with its defective phenotypes. Overall, the prophage-encoded *VpaChn25_0734* (543-bp) gene amplified ecological persistence of *V. parahaemolyticus* CHN25. The results in this study facilitate better understanding of pathogenicity and evolution of *V. parahaemolyticus*, a leading sea foodborne pathogen worldwide.

## Supplementary Information

Below is the link to the electronic supplementary material.

Supplementary file1 (DOCX 16 KB)

## Data Availability

A complete list of the DEGs is available in the NCBI SRA database (http://www.ncbi.nlm.nih.gov/sra/) under the accession number PRJNA733855.
