# Supplementary material for: Prophage-encoded gene VpaChn25_0734 amplifies ecological persistence of Vibrio parahaemolyticus CHN25
Source: Curr Genet. 2022 Jan 22;68(2):267–87. doi: 10.1007/s00294-022-01229-z (PMC8783578; doi:10.1007/s00294-022-01229-z)
Supplement: Supplementary file 1 — Supplementary file1 (DOCX 16 KB) [file 294_2022_1229_MOESM1_ESM.docx]

**Table S1.** Expression of representative DEGs in *ΔVpaChn25_0734* mutant by the RT-PCR assay.

| **Gene** | **Predicted protein** | **Fold change** | |
| --- | --- | --- | --- |
|  |  | **RNA-Seq.** | **RT-PCR** |
| *VpaChn25_RS02080* | outer membrane channel protein TolC | 4.037 | 1.283 |
| *Vpachn25_RS03685* | 4-(cytidine 5'-diphospho)-2-C-methyl-D-erythritol kinase | 2.642 | 2.194 |
| *VpaChn25_RS10950* | flagellar biosynthesis protein FlhF | 4.115 | 3.088 |
| *VpaChn25_RS11345* | isoprenyl transferase | 0.829 | 0.430 |
| *Vpachn25_RS13840* | UTP--glucose-1-phosphate uridylyltransferase GalU | 0.739 | 0.426 |
| *Vpachn25_RS14605* | 6-phosphofructokinase | 0.630 | 0.582 |
